# AI-Powered Spectral Imaging for Virtual Pathology Staining

**DOI:** 10.3390/bioengineering12060655

**Published:** 2025-06-15

**Authors:** Adam Soker, Maya Almagor, Sabine Mai, Yuval Garini

**Affiliations:** 1 Biomedical Engineering Faculty & Russell Berrie Nanotechnology Institute, Technion—Israel Institute of Technology, Haifa 3200003, Israel; yuval.garini@bm.technion.ac.il; 2 Department of Physiology and Pathophysiology, University of Manitoba, Winnipeg, MB R3T 2N2, Canada; sabine.mai@umanitoba.ca

**Keywords:** spectral imaging, virtual staining, digital pathology, artificial intelligence in medicine

## Abstract

Pathological analysis of tissue biopsies remains the gold standard for diagnosing cancer and other diseases. However, this is a time-intensive process that demands extensive training and expertise. Despite its importance, it is often subjective and not entirely error-free. Over the past decade, pathology has undergone two major transformations. First, the rise in whole slide imaging has enabled work in front of a computer screen and the integration of image processing tools to enhance diagnostics. Second, the rapid evolution of Artificial Intelligence has revolutionized numerous fields and has had a remarkable impact on humanity. The synergy of these two has paved the way for groundbreaking research aiming for advancements in digital pathology. Despite encouraging research outcomes, AI-based tools have yet to be actively incorporated into therapeutic protocols. This is primary due to the need for high reliability in medical therapy, necessitating a new approach that ensures greater robustness. Another approach for improving pathological diagnosis involves advanced optical methods such as spectral imaging, which reveals information from the tissue that is beyond human vision. We have recently developed a unique rapid spectral imaging system capable of scanning pathological slides, delivering a wealth of critical diagnostic information. Here, we present a novel application of spectral imaging (SI) for virtual Hematoxylin and Eosin (H&E) staining using a custom-built, rapid Fourier-based SI system. Unstained human biopsy samples are scanned, and a Pix2Pix-based neural network generates realistic H&E-equivalent images. Additionally, we applied Principal Component Analysis (PCA) to the spectral information to examine the effect of down sampling the data on the virtual staining process. To assess model performance, we trained and tested models using full spectral data, RGB, and PCA-reduced spectral inputs. The results demonstrate that PCA-reduced data preserved essential image features while enhancing statistical image quality, as indicated by FID and KID scores, and reducing computational complexity. These findings highlight the potential of integrating SI and AI to enable efficient, accurate, and stain-free digital pathology.

## 1. Introduction

Artificial Intelligence (AI), particularly machine learning (ML), has seen widespread adoption across numerous domains, with healthcare emerging as one of its most impactful arenas. AI technologies have been applied across diverse clinical areas, from improving hematological analysis and managing blood disorders [1] to identifying antibiotic resistance patterns in Mycobacterium tuberculosis [2] and improving time-series predictions through temporal context modeling in dynamic patient monitoring [3]. In medical imaging, AI has been used for automated diagnostics, image segmentation, disease classification, and even prognosis prediction, showing the potential to improve clinical outcomes and reduce human error.

Within the field of pathology, AI has demonstrated remarkable capabilities in detecting cancerous regions, quantifying histological features, and assisting with diagnostic standardization [4,5,6]. These tools hold the potential to significantly enhance efficiency and accuracy in diagnostic pathology. However, despite promising research and technological progress, the clinical integration of AI remains limited due to concerns over reliability, interpretability, and regulatory challenges [7,8].

A major bottleneck in digital pathology is the need for high-quality stained tissue slides, which involves time-intensive sample preparation, chemical reagents, and human expertise. This creates a gap in exploring methods that can eliminate or reduce reliance on physical staining without compromising diagnostic accuracy. Virtual staining, particularly using AI and advanced imaging techniques, offers a potential solution.

In this context, whole slide imaging (WSI) has played a pivotal role. WSI enables the digitization of pathological slides, providing a foundation for AI-based image analysis. It has also facilitated the creation of large, annotated datasets, a key resource for developing ML algorithms. However, standard RGB imaging captures only limited information, motivating the pursuit of richer data modalities.

Spectral imaging (SI) is one such modality, capturing detailed spectral information beyond the capabilities of conventional RGB imaging and revealing subtle molecular and structural differences in tissues. Indeed, a few studies combining SI with digital pathology have demonstrated its potential in the field [9,10,11,12]. However, due to the complexity of SI acquisition methods, these applications have not been extensively explored [13], and their full potential when integrated with AI has yet to be realized. To bridge this gap, we have developed a unique rapid SI system capable of scanning an entire biopsy within a practical time frame for pathological use [14]. Spectral separation relies on Fourier spectroscopy, which utilizes an interferogram placed in the optical path, enabling the simultaneous scanning of spectra for all pixels in the image as described in the Methodology Section. The Fourier method is known for its high throughput advantages, translating to a better signal-to-noise- ratio and shorter acquisition time.

One of the potential applications of SI is virtual staining (VS), where unstained samples are scanned, and AI analysis of the spectra generates color images that mimic traditional stained tissue. This method has two relevant applications. First, VS can generate a color image from an unstained tissue, typically resembling Hematoxylin and Eosin (H&E), the most used stain by pathologists. The second application involves scanning a stained sample, such as H&E, and transforming the image to simulate a different stain, such as MT, Jones [15], or even a fluorescent marker. This capability would provide pathologists with significantly more information than a single stain.

Prima facie, virtual H&E staining may appear unnecessary if the sample is expected to undergo conventional staining, given that H&E remains the gold standard and is used in over 85% [5] of biopsy samples. However, the virtual generation of special stains from H&E images offers significant advantages, as certain histopathological features cannot be identified with H&E alone and typically require additional staining, which is time-consuming, labor-intensive, and dependent on specialized equipment. Training an AI model to convert H&E-stained images into special-stained counterparts, however, presents a major challenge due to the difficulty of obtaining matched pairs of tissue samples with different stains.

A recent paper presented a novel approach for creating registered image pairs of tissue samples stained with H&E and another special stain [5]. The work is based on two networks: the first transforms images of unstained tissue into virtual H&E, and the second transforms H&E images into another special stain. The former model trains on a dataset of unstained and H&E corresponding images of a sample, and the main model trains on the virtual H&E stain resulting from the first model and a matching image of the sample with another special stain. This type of unstained-to-stained AI transformation can be used for in vivo applications, eliminating the need for chemical staining [16].

**Figure 1:**
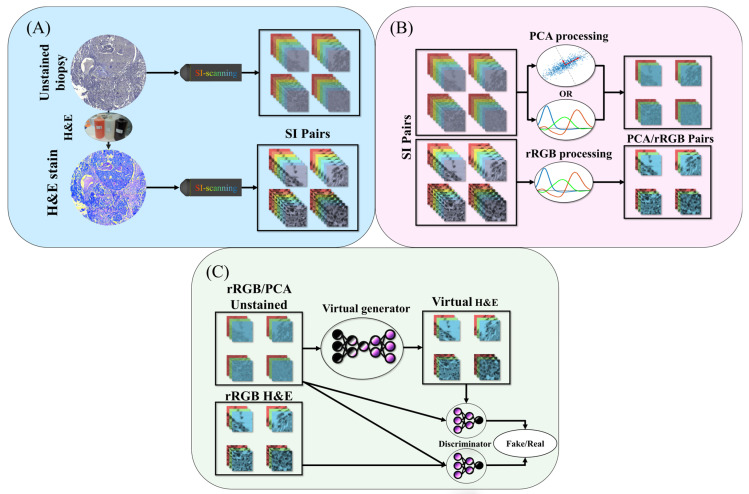
Method workflow. (**A**) Scanning tissue sections with the spectral imaging system, once before the staining process and then again after H&E staining. (**B**) After dividing the images into smaller patches and registering them in pairs, the images are down-sampled to rRGB. Unstained images are also down-sampled using PCA. (**C**) The pairs are used to train supervised Pix2Pix GAN models, once with rRGB and once with PCA, to create images of virtual H&E stain.

In this study, we propose a framework for virtual H&E staining using spectral data from unstained human biopsy samples followed by AI analysis (Figure 1). We used breast cancer biopsy samples that were scanned by our SI system twice, first without any stain, and again following standard H&E staining protocol (Figure 1A). The spectral images of the stained dataset went through a process of reduction to Red–Green–Blue (rRGB) based on the CIE 1931 scheme [17]. The unstained dataset was also reduced to rRGB (Figure 1B), but in addition, it was reduced by Principal Component Analysis (PCA) [18]. In contrast to previous works on virtual H&E staining, we did not make use of autofluorescence [5,19] in the invisible range; instead, we measured only the bright-field transmission in the visible spectral range. This is simpler and faster in comparison to fluorescence measurements, and yet, we achieved high performance, emphasizing the applicability of spectral information.

The VS model is based on the Pix2pix model [20] (Figure 1C) and was trained several times with different numbers of PCA components, with rRGB, and with the full spectrum. For all pre-processing methods, the results had comparable spatial metrics such as L1 [20], RMSE [21], PNSR [22], and SSIM [23], with a minor advantage for PCA with 10 and 11 elements. However, when we evaluated the results with statistical measures such as FID [24] and KID [25], PCA with five components outperformed all other tests, including the full spectrum and rRGB.

## 2. Methodology

This study follows a multi-stage process that integrates SI with deep learning for virtual staining of pathology samples. First, we acquired spectral images of breast cancer biopsy tissues using our custom-developed rapid Fourier-based SI system, capturing data from each sample both before and after H&E staining. Next, we preprocessed the spectral data by reducing dimensionality through Principal Component Analysis (PCA) and converting images to Red–Green–Blue (rRGB) representations. These datasets were manually registered and then used to train multiple Pix2Pix conditional GAN models for virtual staining. We evaluated model performance across different preprocessing methods using both spatial (L1, RMSE, SSIM, and PSNR) and statistical (FID and KID) metrics. Finally, we evaluated the quality of the data with an expert. This section outlines these stages, including SI system and data acquisition, datasets preparation, network architecture design, and dimensionality reduction.

### 2.1. Spectral Imaging System

**Figure 2:**
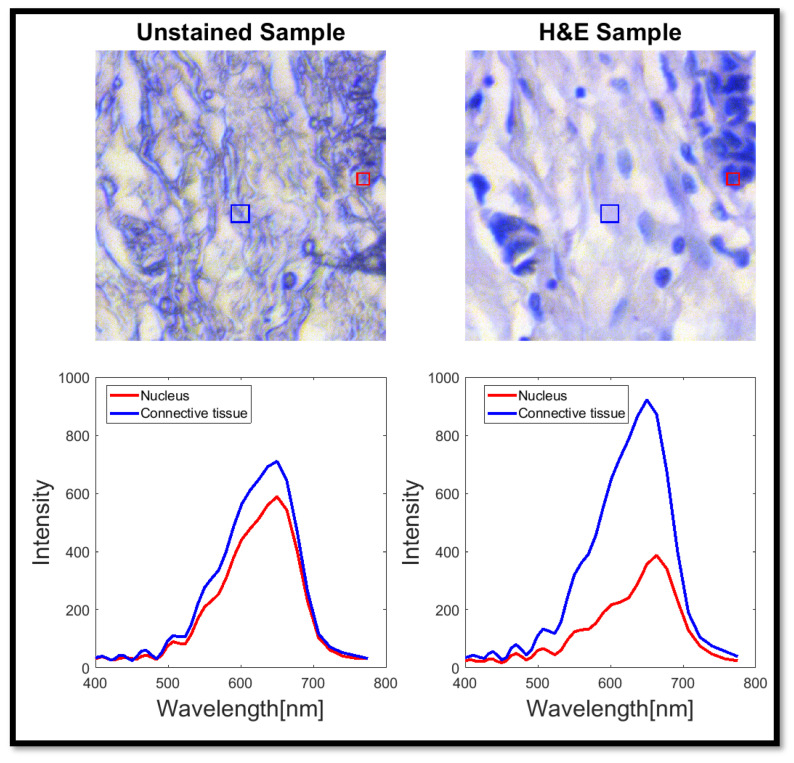
Example of nuclei and connective tissue spectra from images of an unstained sample (**left**) and an H&E-stained sample (**right**). Note that the unstained tissue is highly transparent and reveals very few details. To improve its visibility, we applied a color enhancement that renders the image bluish and somewhat artificial appearance, while emphasizing the spatial details of the tissue. The bottom graphs show the average transmission spectra from the blue and red squares shown in the images. The images area is $141\times 141\;[{\mu m}^{2}]$ as measured with a 20× magnification.

Unlike traditional RGB imaging, which assigns three values per pixel, a spectral image captures a full spectrum for each pixel, typically consisting of 10–100 different wavelengths (see examples of 40 wavelengths in Figure 2). This results in a three-dimensional data structure that is widely used across various fields, including remote sensing, agriculture, and even art preservation [13]. Previous studies have highlighted the significance of SI in various life sciences applications [9,19,26], particularly in genetic screening through spectral karyotyping (SKY) [27,28]. However, its use in pathology remains limited, despite its potential to enhance AI applicability for clinical practice. One major challenge of SI is its relatively long acquisition time due to the large volume of data collected compared to standard RGB imaging. Most existing SI methods rely on a set of color filters matched to specific wavelength ranges, where the number of spectral points is determined by the number of filters used.

**Figure 3:**
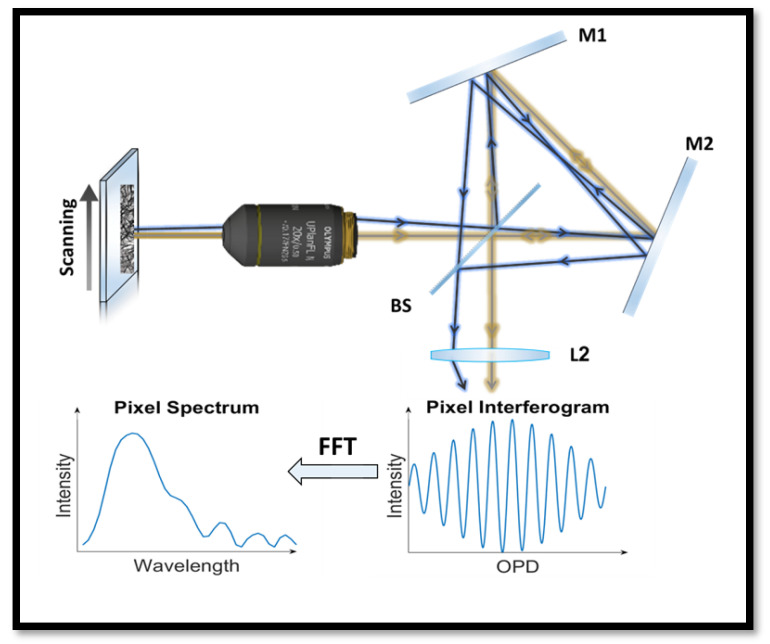
SI system scheme. The collimated beam emerging from the microscope objective lens passes through the Sagnac interferometer, where an optical path difference is introduced for each beam based on its entrance angle, as illustrated by blue and yellow ray traces. At the exit from the interferometer, a lens focuses the light onto a CMOS camera. As the sample is scanned, an interferogram is recorded at each pixel. These interferograms are then Fourier-transformed to retrieve the corresponding spectra, yielding a full spectrum for each pixel in the image.

Lately we developed a spectral imaging system based on Fourier spectroscopy [14], that measures the spectrum indirectly by measuring an interference pattern known as the interferogram for each pixel (Figure 3). Each interferogram, captured for each pixel in the image, represents intensity as a function of optical path difference (OPD) generated by a Sagnac interferometer. These interferograms are collected simultaneously for all the pixels in the image (Figure 3). The system has no moving parts, except for a microscope computer-controlled stage that is needed for scanning the sample anyway, similarly to WSI systems [29].

The spectrum at every pixel is derived from the interferogram using a fast Fourier transform along with well-established pre- and post-processing steps [30]. These operations are standard in Fourier spectroscopy and include the following: 1. apodization, which removes high-frequency artifacts in the spectrum arising from the finite length of the interferogram; 2. zero-filling, which better emphasizes the spectral resolution by interpolating additional data points; and 3. phase correction, which converts the complex spectra obtained after the Fourier transform into real-valued spectra. A detailed description of these common procedures can be found in Lindner et al. [14].

Biopsy slides are measured on an Olympus IX81 inverted microscope equipped with a motorized stage (Prior Scientific, Cambridge, UK). The samples are measured with a 20× objective lens with NA = 0.8. A typical spectrum consists of 40 points in the visible spectral range of 400–750 nm. The spectral resolution in Fourier spectroscopy changes along the spectral range from 5 nm at 400 nm to 20 nm at 800 nm [14]. The CMOS camera (Lumenera Lt225 NIR, now Teledyne Lumenera, Ottawa, ON, Canada) has a pixel size of 5.5 × 5.5 μm^2^ so that each pixel images a sample area of 275 × 275 nm^2^. This is oversampling as the spatial resolution is limited to ~610 nm by the diffraction limit. The acquisition occurs ‘on the fly’ while the stage moves at a constant speed and the camera measures 150 images/second with an exposure time of 10 μs. Although the stage continues to move during camera exposure, its speed is carefully selected to ensure that the image smear remains below a quarter of a pixel in size, thereby preserving the spatial resolution. The system can also operate in ‘stop-and-go’ mode, which extends the acquisition time and is typically used when imaging low-light conditions that require longer exposure times.

At the end of the acquisition process, the recorded intensities at each sample point are assembled to construct the interferogram at every pixel. The performance of the system was previously evaluated and shown to achieve high-quality imaging, limited by the diffraction limit [14]. Further details are provided in the Supplementary Materials.

### 2.2. Sample Preparation

Pathology tissue array slides were provided by TissueArray (Derwood, MD, USA) and include 9 cases of breast cancer in patients of different ages (44–58). Each case included 1–2 paraffin-embedded tissue samples, 5 μm thick and approximately 1.5 mm in diameter. Each sample was scanned twice, once before staining and again after H&E staining using the ab245880 H&E Kit (Abcam, Cambridge, UK). The main steps included deparaffinization and hydration of the sections, spectral imaging (SI) scanning, staining with Hematoxylin, rinsing with distilled water, brief application of Bluing Reagent, a second rinse, dipping in absolute alcohol, staining with Eosin Y solution, rinsing with alcohol, dehydration through graded alcohols, clearing, mounting in synthetic resin, and final rescanning.

The registration of unstained and stained images of the same tissue was performed manually using a Graphical User Interface (GUI) that we developed (MATLAB Version R2021b). To simplify the procedures, small 256 × 256 pixel images were measured from each biopsy pair with 50% overlap. Each image corresponds to a sample area of ~70 × 70 ${\mu m}^{2}$, and each tissue sample typically yielded 1000 to 2000 small images. For model development, we assembled a training dataset comprising 13,000 images from six different patients, and a test dataset containing 5000 samples from three different patients.

### 2.3. Principal Component Analysis

Principal Component Analysis (PCA) is employed as a data preprocessing technique to reduce the dimensionality of the spectral information before it is fed into the deep learning model. PCA is a widely utilized statistical technique in data analysis that facilitates a reduction in the dimensionality of datasets while preserving as much variance of data as possible [18]. This reduction is achieved by transforming the original variables into a new set of uncorrelated variables known as principal components, ordered by the amount of variance they capture from the data. The core idea behind PCA is to identify the directions along which the variation in the data is maximized. These directions are orthogonal to each other, ensuring that the principal components are linearly independent.

Mathematically, PCA involves covariance matrix computation, which is then decomposed into its eigenvalues and eigenvectors, representing the directions and the magnitude of the principal components, respectively. The original data is projected onto the eigenvectors to form the principal components, ranked according to their variance. By selecting the top n components, a reduced-dimensionality representation of the data is obtained. Another perspective on PCA is that it reduces the dimensionality of a dataset while preserving as much relevant information as possible. The loss is defined as

(1) $$Loss\left(n\right)=\frac{{\left\|x_{i}-{\hat{x}}_{i}\left(n\right)\right\|}_{2}^{2}}{{\sum_{i=1}^{m}\|x_{i}\|}_{2}^{2}};\;{\hat{x}}_{i}\left(n\right)=\sum_{j=1}^{n}p_{i,j}v_{j}$$

where $x_{i}$ (resized to one-dimensional) is the real spectral image, ${\hat{x}}_{i}\left(n\right)$ is the reconstruction image of $x_{i}$, $v_{j}$ is the PC vector, $p_{ij}$ is the $x_{i}$ projection on $v_{j}$, and n is the size of the reduced dimension. Because ${\hat{x}}_{i}\left(n\right)$ is a projection of $x_{i}$ onto a reduced dimension, this can be considered a loss of information. It means that a perfect reconstruction gives a $\mathrm{Loss}\left(n\right)$ that equals zero. The PCA solutions are given by the eigenvectors of the covariance matrix:

(2) $$C=\frac{1}{m-1}{\bar{X}}^{T}\bar{X};\;Cv_{j}=\lambda_{j}v_{j};\;\bar{X}=\;\left(\overset{|}{\underset{|}{x_{1}}},\overset{|}{\underset{|}{x_{2}}}\ldots \overset{|}{\underset{|}{x_{m}}}\right)-\sum_{j=1}^{n}{\hat{x}}_{j}\;$$

where m is the sample number, $\lambda_{i}$ are the sorted eigenvalues ($\forall p>q:\;\lambda_{p}>\lambda_{q}$) corresponding to the eigenvectors $v_{j}$, and $\bar{X}$’s columns are the centralized original samples. In this case Equation (3) is given as follows:

(3) $$Loss\left(n\right)=1-\frac{\sum_{i=1}^{n}\lambda_{j}}{\sum_{i=1}^{N}\lambda_{j}}$$

where N is the size of the full spectrum (*N* = 40).

An additional benefit of PCA is that noises with low standard deviations do not affect the lower dimensions [31]. As each spectrum contains a high dimension of 40 spectral channels, it can be computationally expensive and potentially redundant. Therefore, our PCA approach that was applied to the unstained spectral images before model training allowed us to assess the impact of the reduced spectral representation on the quality of virtual H&E staining. PCA was applied to reduce the number of spectral channels to 3–15 candidate components, and the minimum number required to preserve high-quality virtual staining performance was determined. The impact of this dimensionality reduction on both spatial and statistical image quality metrics was subsequently analyzed to assess its effectiveness for spectral-to-stain image translation.

### 2.4. Network Architecture

To generate stained tissue images from spectral data of unstained tissue sections, we employed a conditional Generative Adversarial Network (GAN) based on the Pix2Pix framework, designed for image-to-image translation, originally introduced by Phillip Isola et al. [20]. The network is trained to map input images to output images, learning to generate a realistic image conditioned on an input image. A conditional GAN (cGAN) is a type of GAN where the generator and discriminator are conditioned on some additional information. The generation process is guided by this input, allowing for more controlled and specific outputs. In our case, the input to the network is a spectral image of an unstained tissue section, and the target is its corresponding H&E-stained image. The generator is trained to produce realistic stained images that closely match the ground truth, while the discriminator learns to distinguish between real and generated (virtual) stains.

**Figure 4:**
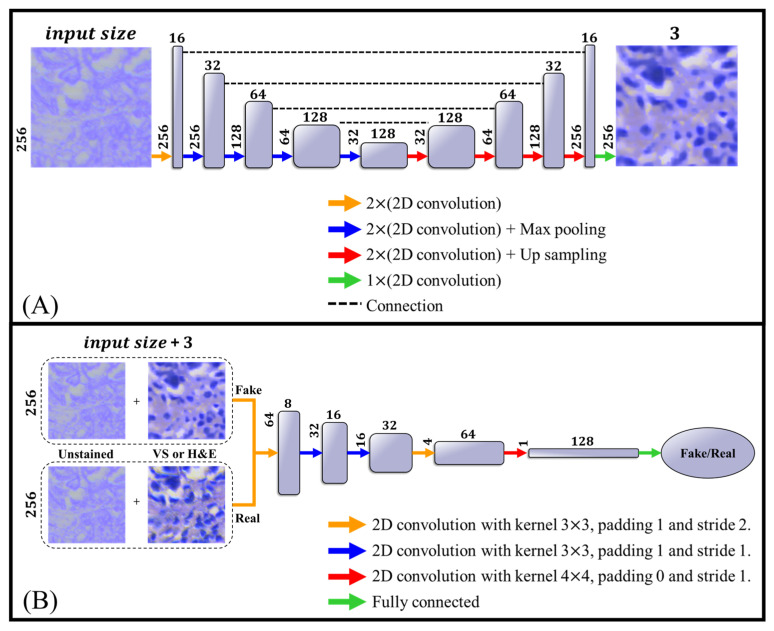
Model architecture. (**A**) The generator model, which is based on U-net. All convolutions had kernels with a size of 3 × 3 and included batch normalization and a LeakyReLU [34] function with a parameter of 0.1. (**B**) The discriminator model, based on the Patch model. Here too, all convolutions included batch normalization and a LeakyReLU function with a parameter of 0.1.

The generator in Pix2Pix adopts a U-Net architecture [32] (Figure 4A), which consists of an encoder–decoder structure with skip connections between corresponding layers. These skip connections are essential for preserving spatial resolution and fine structural details during the translation process. Each convolutional layer uses 3 × 3 kernels, followed by batch normalization and a LeakyReLU activation function with a negative slope of 0.1.

The discriminator is implemented as a PatchGAN (Figure 4B), which classifies local image patches as real or fake, rather than the entire image. This design encourages the generator to produce realistic texture and fine-grained details. Like the generator, all convolutional layers in the discriminator include batch normalization and LeakyReLU activations (slope = 0.1).

Pix2Pix uses a combination of GAN loss and similarity loss. The former is calculated as the extent to which the generator succeeds in ‘fooling’ the discriminator and thereby helps in generating realistic images, while the latter ensures that the generated images closely resemble the ground truth (GT) images at a pixel level. The similarity loss is computed as the L1 loss between the GT and the virtually stained (VS) images, following the application of a Gaussian blur (kernel size 5 × 5) to both, to emphasize overall structural features over pixel-level noise. The generator loss can be defined as

(4) $${L_{G}}_{\left(x,y\right)\sim \left(p_{X,Y}\right)}\left(G\left(x\right),D,y,x\right)=\log\left(D\left(G\left(x\right),y\right)\right)+\gamma_{1}{\left\|\hat{G}\left(x\right)-\hat{y}\right\|}_{1}+\gamma_{2}TV\left(x\right)$$

where G and D are the generator and discriminator, $P_{X,Y}$ is the GT dataset of unstained and H&E pairs, $\gamma_{1}$ and $\gamma_{2}$ are hyper-parameters equal to 500 and $10^{-4}$, respectively, $\hat{G}\left(x\right)$ and $\hat{y}$ are the modified $G\left(x\right)$ and y after the Gaussian blur, and $\mathrm{TV}\left(x\right)$ is a total variation regulation term [33], given by

(5) $$TV\left(x\right)=\sum_{c=1}^{3}\sqrt{\sum_{i=1}^{H-1}\sum_{j=1}^{W}{\left(x_{i,j,c}-x_{i+1,j,c}\right)}^{2}+\sum_{i=1}^{H}\sum_{j=1}^{W-1}{\left(x_{i,j,c}-x_{i,j+1,c}\right)}^{2}}$$

where H and W are the spatial dimensions, and c is the image color. This regulation enhances the quality of realistic images, as natural images typically exhibit low variance, resulting in smooth and coherent images.

Like all GANs, we train the discriminator in parallel using the following loss function:

(6) $${L_{D}}_{\left(x,y\right)\sim \left(p_{X,Y}\right)}\left(G\left(x\right),D,y,x\right)=\frac{1}{2}\log\left(D\left(x,y\right)\right)-\frac{1}{2}\log\left(D\left(G\left(x\right),y\right)\right)$$

This formulation ensures balanced adversarial training by encouraging the discriminator to distinguish between real and generated pairs while pushing the generator toward more convincing outputs. Changes in the loss function throughout the training process are shown in the Supplementary Material.

## 3. Results

### Model Training

The model was trained multiple times using varying numbers of PCA components ranging from 1 to 15 and 20, as well as with the full SI data and rRGB inputs. For all methods, the datasets were normalized by centering and scaling each dimension separately to the average and STD of all the training databases [35]. Model parameters optimization was performed using the Adam optimizer [36] over 100 epochs, with the learning rate initially set to 0.02 and decayed by a factor of 0.85 every five epochs. All training processes were conducted on an NVIDIA GeForce RTX 3090 GPU (NVIDIA, Santa Clara, CA, USA).

**Figure 5:**
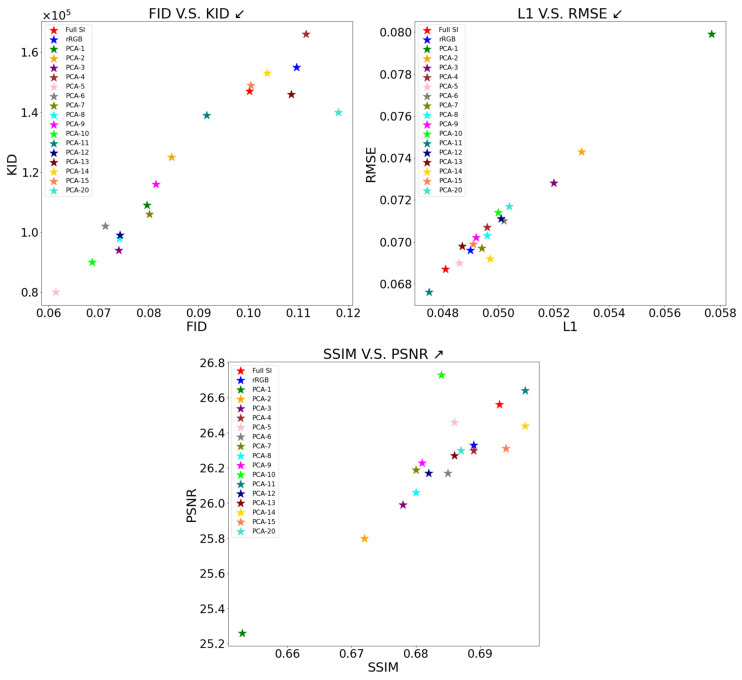
The six metrics for all types of datasets are displayed across three different scatter plots. All dataset methods achieved similar results in the spatial metrics, including L1, RMSE, PNSR, and SSI, with a small advantage for PCA-10 and PCA-11. However, the evaluation of the results using statistical measures (FID and KID) shows a clear advantage for PCA-5. Arrows on the figure titles indicate the direction of better results.

**Figure 6:**
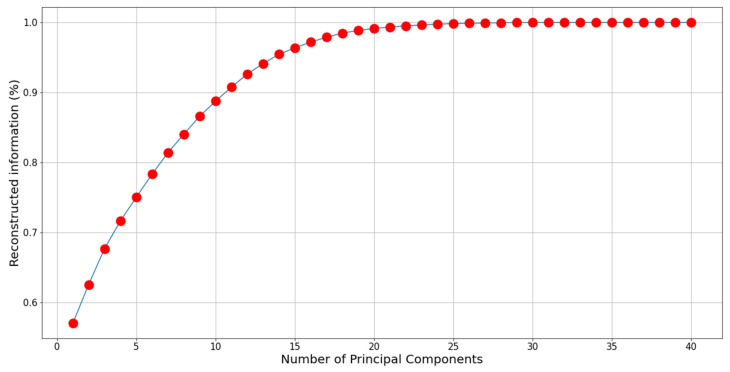
Reconstruction information of 1-Loss(n) as a function of the number of components. Notably, the first component captures over 50% of the total information, while the first 5 components account for 75% of the information.

As one can see in Table 1 and Figure 5, all dataset methods achieved similar results in the spatial metrics, including L1, RMSE, PNSR, and SSI, with a small advantage for PCA with 10 and 11 elements. However, the evaluation of the results using statistical measures (FID and KID) shows a clear advantage for PCA with five elements. These results are consistent with Figure 6, which shows information loss per number of PCA components, where the loss is defined as $1-\mathrm{Loss}\left(n\right)$ (Equation (3)). As shown in Figure 6, using PCA with five components preserves approximately 75% of the original information. Despite information loss, the PCA-reduced data achieve high performance due to lower dimensionality and random noise cleaning [37,38].

**Figure 7:**
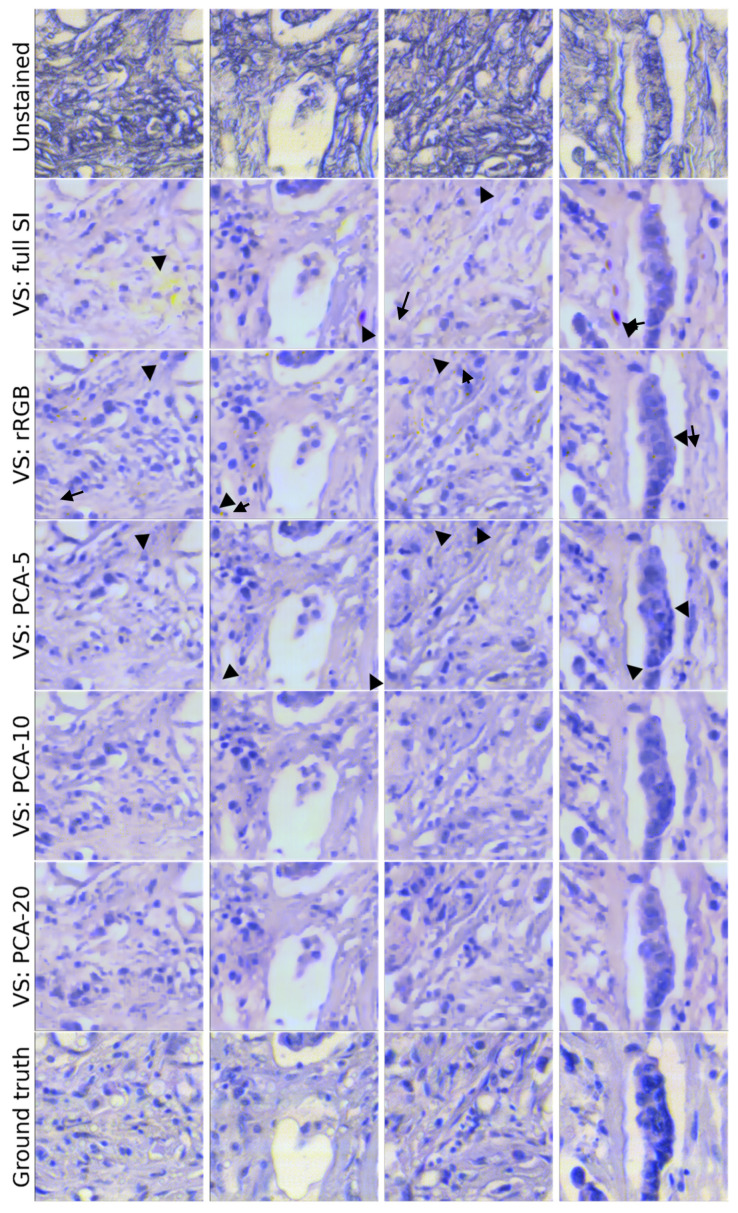
Examples of images reconstructed from unstained tissues using different models. The reconstructions based on full SI and rRGB exhibited higher noise levels and reduced effectiveness in identifying nuclei compared to results obtained using PCA. In addition, the VS method presented a cleaner result than the GT method. Each image is size is $141\times 141\;[{\mu m}^{2}]$ as measured with a 20 × magnification. The black arrows indicate inaccuracies of the full SI- and rRGB-based models compared to the PCA-5 based model.

In Figure 7 the results of four cases (images) are presented, including an unstained sample, an H&E-stained sample, and the results from five models: three based on PCA (with five, ten, and twenty components), one based on rRGB, and one using the full spectrum. The results show that the model using the full spectrum mimics the stained tissue across the image with high accuracy. Nevertheless, it suffers from a few artifacts, particularly in bright regions. Additionally, both the full-spectrum and rRGB-based models failed to detect some nuclei and, in some cases, hallucinated nuclei that do not exist.

Based on an expert assessment, there is a high level of concordance between the commonly used Hematoxylin/Eosin (H&E) staining of the tissue and the ‘fake’ staining that was generated by AI. This is also supported by the statistical analysis presented above.

We hypothesize that the spatial metrics are limited because of structural changes in the staining process and artifacts, as one can see in Figure 7, which prevent high performance of the model without overfitting in the training set. This fact can explain the comparable spatial metric values. However, these models showed high-quality results, demonstrating the advantage of using SI data over RGB data. We assume that using cleaner data and autofluorescence information could improve the results even more.

## 4. Discussion

Although virtual H&E staining holds considerable promise for medical applications, further in-depth research is still required. To date, SI-based studies have been relatively scarce, primarily due to the technique’s inherent complexity and lengthy acquisition times. In this work, we present an innovative SI system based on Fourier spectroscopy that provides full spectral resolution at the 10 nm level while significantly shortening acquisition time, overcoming a major limitation of previous methods.

Previous studies have demonstrated the advantages of incorporating spectral information into AI-driven applications such as nucleus segmentation [9], cancer cells segmentation [10,39], and cancer detection [26,40,41]. Several works have also highlighted the value of SI in VS applications [19,42], as well as the utility of PCA in processing spectral data [43]. However, the extended acquisition time required for SI has remained a major limitation, often necessitating a compromise between data volume and spatial resolution. Fourier spectroscopy-based systems address this challenge by enabling the use of SI without these compromises.

A major challenge in SI that remains is the vast amount of data, much of which may be irrelevant, along with the noise introduced during acquisition. To address this, we evaluated a supervised AI-based VS approach using PCA for spectral dimensionality reduction. PCA serves as an effective down-sampling technique, while preserving essential information and simultaneously reducing noise.

The rapid SI system and its AI-based analysis have demonstrated excellent performance, surpassing both state-of-the-art results based on RGB information and results based on the full spectrum. This advantage persists even when one-dimensional PCA is utilized, further highlighting the effectiveness of the method. While the improvement in spatial metrics was minor, likely due to limitations caused by morphological differences between the unstained tissue and the H&E-stained tissue, a noteworthy improvement in statistical metrics was observed, with the highest result obtained for five spectral components.

We hypothesize that this behavior stems from the finite set of biological constituents that make up the tissue, each associated with characteristic absorption and transmission spectra. Consequently, the measured spectral data can be effectively represented by a finite number of components.

Accordingly, the integration of spectral imaging with AI and PCA in the analysis of pathological samples offers a rich source of information that is doable, practical, and able to enhance diagnostic accuracy in pathology.

Several points remain to be elucidated in future studies, including the following:

1. There is a difference in the morphology of unstained and stained tissue irrespective of ‘fake’ or true H&E staining. This is most obvious for areas without cells that are found throughout tissue (see Figure 7); such areas became smaller and changed their shapes after H&E staining. Considering this observation, other morphologies must change as well but are not as obvious to the eye. Such changes after H&E staining are not currently known in the field. Thus, further studies will need to be performed to define the staining-induced differences in morphology and to determine whether they matter in clinical diagnosis.
2. While the concordance between real H&E and ‘fake’ H&E is high (Table 1), ‘fake’ H&E appears to sometimes add ‘stained’ structures (looking like cells) or fails to identify some cells that were stained by H&E. Further studies need to investigate the reason for this difference.
3. It is expected that these minor differences between ‘fake’ H&E and true H&E staining should not impact the diagnostic value of the ‘fake’ H&E. It is important to perform a blind study that will compare a series of patient samples examined after fake H&E and true H&E staining. If the examination of such samples by a pathologist leads to identical results in diagnosis, the minor differences reported in this study should not matter for clinical evaluation.

## 5. Conclusions

Our results highlight the significance of incorporating spectral information beyond standard RGB data. By leveraging such methods, a biopsy stained with one staining method could yield information equivalent to that of another or even multiple staining methods. This approach has the potential to enhance diagnostic accuracy, providing pathologists with critical insights while eliminating the need for complex and time-consuming multiple-staining procedures.

Furthermore, here we used only the visible light range (400–700 nm) for spectral measurements. Nevertheless, we believe that also incorporating the invisible light spectrum, especially in the infrared spectral range, or further physical information, such as polarization and Raman scattering, could further improve the results.

The integration of spectral imaging with AI presents a transformative opportunity for the field of pathology. By enabling stain-free or stain-to-stain analysis, this approach has the potential to streamline diagnostic workflows, improve accuracy, and reduce turnaround times. The ability to extract rich biochemical and structural information from native tissue further enhances diagnostic sensitivity and specificity. Ultimately, these advancements not only support more precise and efficient pathology practices but also contribute to earlier disease detection and improved patient outcomes, marking a significant step forward in global healthcare.

## Figures and Tables

**Table 1:** Summary of the performance of selected methods across six different evaluation metrics. The models were trained using the full spectral information, RGB-only reduction, and various numbers of principal components (5,10,15 and 20). Bold values indicate the best performance for each metric. The arrows next to the metrics names indicate the direction of better results. A down-pointing arrow means that a smaller number relates to a better performance and vice versa.

Dataset	FID↓	KID↓	L1↓	RMSE↓	SSIM↑	PSNR↑
**Full SI**	0.1002	$147\cdot 10^{3}$	0.0481	0.0687	0.693	26.56
**rRGB**	0.1096	$155\cdot 10^{3}$	0.0490	0.0696	0.689	26.33
**PCA-5**	0.0614	$80\cdot 10^{3}$	0.0486	0.0690	0.686	26.46
**PCA-10**	0.0687	$90\cdot 10^{3}$	0.0500	0.0714	0.684	26.73
**PCA-15**	0.1005	$149\cdot 10^{3}$	0.0491	0.0699	0.694	26.31
**PCA-20**	0.1180	$140\cdot 10^{3}$	0.0504	0.0717	0.687	26.30

## Data Availability

Due to the unique nature and complexity of the hyperspectral imaging data used in this study, we have opted to make the dataset available upon request. This approach ensures that we can provide appropriate guidance regarding the data’s structure, acquisition process, and intended use. Researchers interested in accessing the data are welcome to contact the corresponding authors at adam-soker@campus.technion.ac.il or yuval.garini@bm.technion.ac.il.

## Supplementary Materials

The following supporting information can be downloaded at: https://www.mdpi.com/article/10.3390/bioengineering12060655/s1, Table S1: The table of all the wavelengths that the system has detected; Figure S1: All the wavelengths according to their serial number; Figure S2: Similarity loss for all methods throughout the training stages (every 5 epochs).
