# Supplementary material for: AI-Powered Spectral Imaging for Virtual Pathology Staining
Source: Bioengineering (Basel). 2025 Jun 15;12(6):655. doi: 10.3390/bioengineering12060655 (PMC12189724; doi:10.3390/bioengineering12060655)
Supplement: Supplementary file 1 [file bioengineering-12-00655-s001.zip › bioengineering-3562563-supplementary.pdf]

Supplement

Spectral characteristics

As described in the *Methodology* section, an initial calibration was performed to determine the appropriate spectral range and resolution for the FFT-based measurement method. Table lists the exact wavelengths measured and Figure shows the wavelengths according to their serial number.
It is important to note that the wavelengths calculated using the FFT are not evenly spaced. Consequently, the spectral resolution increases with wavelength, ranging from approximately 5 nm at λ = 400 nm to around 17 nm at λ = 770 nm.

Table S1
The table of all the wavelengths that the system has detected

Ordinal number	1	2	3	4	5	6	7	8	9	10
wavelength[nm]	400	405	410	415	421	426	432	438	444	450
Ordinal number	11	12	13	14	15	16	17	18	19	20
wavelength[nm]	456	463	470	477	484	491	499	507	515	523
Ordinal number	21	22	23	24	25	26	27	28	29	30
wavelength[nm]	532	541	550	560	569	580	590	601	613	625
Ordinal number	31	32	33	34	35	36	37	38	39	40
wavelength[nm]	637	650	663	677	692	707	723	739	757	775

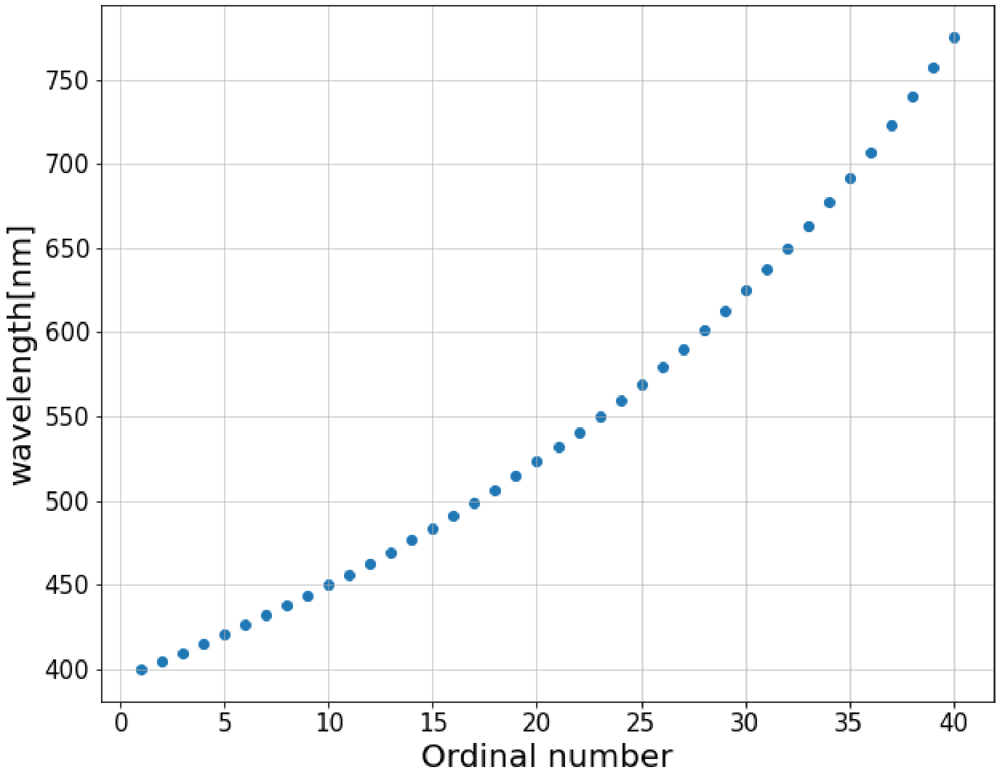

Figure S1
All the wavelengths according to their serial number.

Loss function

The similarity loss function was measured for all methods throughout the training stages (every 5 epochs) and are presented in Figure

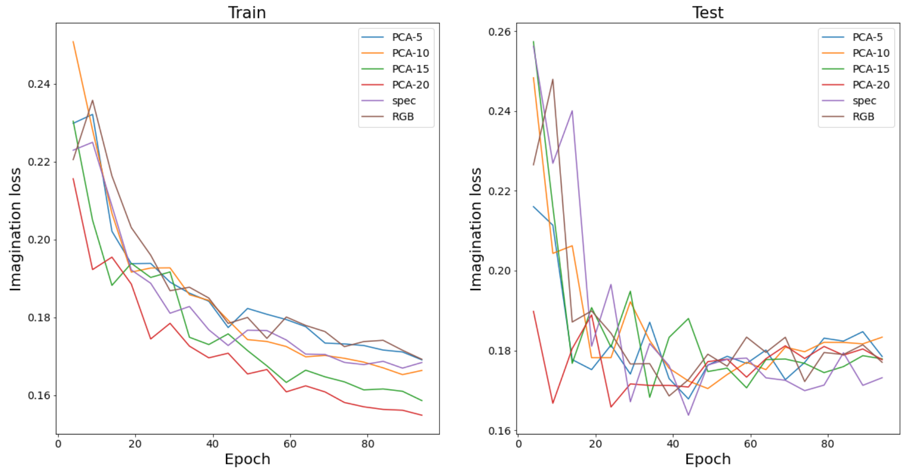

Figure S2
similarity loss for all methods throughout the training stages (every 5 epochs).

H&E Stain Kit (Hematoxylin and Eosin)

ab245880– H&E Stain Kit (Hematoxylin and Eosin)

For use in histology and cytology applications

View Datasheet: www.abcam.com/ab245880 [use www.abcam.cn/ab245880 for China, or www.abcam.co.jp/ab245880 for Japan)

This product is for research use only and is not intended for diagnostic use

Overview

H&E Stain Kit (Hematoxylin and Eosin) ab245880 is intended for use in histology and cytology applications. Included in this kit is a newly formulated Eosin that provides the benefits of a traditional alcoholic formula with significant improvements in usability. Advantages include lower evaporation rate, better color patterns, reduced tendency to spill over container, hands, and countertops, and improved surface tension to remain on tissue section. Our Hematoxylin produces crisp, intense blue nuclei providing optimal contrast to the Eosin stained cytoplasm.

Staining Interpretation:

Cytoplasm: Light pink
Collagen: Pink
Muscle: Pink/Rose
Erythrocytes: Pink/Red
Nuclei: Blue

Control Tissue:

Any well fixed paraffin embedded or frozen tissue section. Cell smear.

Precautions

Please observe safe laboratory practice and consult the safety datasheet.

For general guidelines, precautions, limitations on the use of our assay kits and general assay troubleshooting tips, particularly for first time users, please consult our guide: www.abcam.com/assaykitguidelines

For typical data produced using the assay, please see the assay kit datasheet on our website.

Materials Supplied:

Store kit at Room temperature immediately on receipt and check below for storage for individual components. Kit can be stored for 1 year from receipt, if components have not been reconstituted.

Keep away from open flame and refer to the safety datasheet.

Item	Quantity	Storage temperature (before prep)	Storage temperature (after prep)
Hematoxylin, Mayer's (Lillie's Modification)	500 mL	RT	RT
Bluing Reagent	500 mL	RT	RT
Eosin Y Solution (Modified Alcoholic)	500 mL	RT	RT

Assay Procedure

Equilibrate all materials and prepared reagents to room temperature just prior to use and gently agitate.

1. Deparaffinize sections if necessary and hydrate in distilled water.
2. Apply adequate Hematoxylin, Mayer's (Lillie's Modification) to completely cover tissue section and incubate for 5 mins.
3. Rinse slide in two changes of distilled water to remove excess stain.
4. Apply adequate Bluing Reagent to completely cover tissue section and incubate for 10-15 secs.
5. Rinse slide in two changes of distilled water.
6. Dip slide in absolute alcohol and blot excess off.
7. Apply adequate Eosin Y Solution (Modified Alcoholic) to completely cover tissue section to excess and incubate for 2-3 mins.
8. Rinse slide using absolute alcohol.
9. Dehydrate slide in three changes of absolute alcohol.
10. Clear slide and mount in synthetic resin

Technical Support

Copyright © 2020 Abcam. All Rights Reserved. The Abcam logo is a registered trademark. All information / detail is correct at time of going to print.

For all technical or commercial enquiries please go to:
www.abcam.com/contactus
www.abcam.cn/contactus (China)
www.abcam.co.jp/contactus (Japan)

Tissues details

BR245b Breast cancer with adjacent normal breast tissue array (2012 WHO classification), including pathology grade, TNM and clinical stage (A.

12 cases/24 cores, replaced by BR246d, unstained slide

Microarray Panel	Breast carcinoma with adjacent normal breast tissue microarray, containing 10 cases of invasive carcinoma of no special type, 2 adjacent normal breast tissue, duplicate cores per case
Cores	24
Cases	12
Row number	4
Column number	6
Core Diameter (mm)	1.5
Thickness (μm)	5
Tissue Array Type	FFPE
Species	Human
Applications	Routine histology procedures including Immunohistochemistry (IHC) and In Situ Hybridization (ISH), protocols which can be found at our support page.
Notes	1. TMA slides were sectioned and stored at 4°C and may not be fresh cut, but still suitable for IHC. Please request fresh cut if experiment involves phospho-specific antibodies, RNA studies, FISH or ISH, etc. A minimum of 3 slides per TMA must be purchased to cover the cost of trimming for fresh sectioning. 2. Most TMA slides were not coated with an extra layer of paraffin (tissue cores can be easily seen on the glass). **To prevent tissue detachment during antigen retrieval, unbaked slides must be baked for at least 30 to 120 minutes at 60°C.** before putting into xylene for de-paraffinization. Baked slides were sent out baked for 2 hours.<br>In the following specsheet,"*" means invalid core; "-" means no applicable or negative in IHC markers.

BR245b H&E

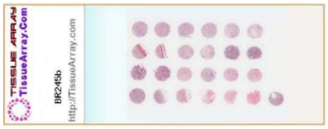

Mouseover and click individual cores to view high resolution images.

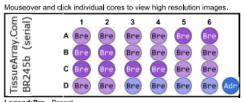

Legend:Bre - Breast
- Malignant tumor, - Malignant tumor (stage IIA), - Malignant tumor (stage IIB), - Malignant tumor (stage IIIA), - Malignant tumor (stage IIIB),
- NAT

, tissue IDs are available in exported Excel files.

Pos.	No.	Age	Sex	Organ/Anatomic Site	Pathology diagnosis	TNM	Grade	Stage	Type	Image
A1	1	52	F	Breast	Invasive carcinoma of no special type	T2N0M0	2	IIA	Malignant	
A2	2	52	F	Breast	Invasive carcinoma of no special type	T2N0M0	2	IIA	Malignant	
A3	3	50	F	Breast	Invasive carcinoma of no special type	T2N1M0	2	IIB	Malignant	
A4	4	50	F	Breast	Invasive carcinoma of no special type	T2N1M0	2	IIB	Malignant	
A5	5	49	F	Breast	Invasive carcinoma of no special type	T3N1M0	2	IIIA	Malignant	
A6	6	49	F	Breast	Invasive carcinoma of no special type	T3N1M0	2	IIIA	Malignant	
B1	7	55	F	Breast	Invasive carcinoma of no special type	T4N2M0	2	IIIB	Malignant	
B2	8	55	F	Breast	Invasive carcinoma of no special type	T4N2M0	2	IIIB	Malignant	
B3	9	44	F	Breast	Invasive carcinoma of no special type	T2N0M0	2	IIA	Malignant	
B4	10	44	F	Breast	Invasive carcinoma of no special type	T2N0M0	2	IIA	Malignant	
B5	11	58	F	Breast	Invasive carcinoma of no special type	T2N1M0	2	IIA	Malignant	
B6	12	58	F	Breast	Invasive carcinoma of no special type	T2N1M0	2	IIA	Malignant	
C1	13	30	F	Breast	Invasive carcinoma of no special type	T2N2M0	2	IIIA	Malignant	
C2	14	30	F	Breast	Invasive carcinoma of no special type	T2N2M0	2	IIIA	Malignant	
C3	15	35	F	Breast	Invasive carcinoma of no special type	T2N2M0	2	IIIA	Malignant	
C4	16	35	F	Breast	Invasive carcinoma of no special type	T2N2M0	2	IIIA	Malignant	

C5	17	54	F	Breast	Invasive carcinoma of no special type	T2N1M0	2	IIB	Malignant	
C6	18	54	F	Breast	Invasive carcinoma of no special type	T2N1M0	2	IIB	Malignant	
D1	19	43	F	Breast	Invasive carcinoma of no special type	T2N1M0	3	IIB	Malignant	
D2	20	43	F	Breast	Invasive carcinoma of no special type	T2N1M0	3	IIB	Malignant	
D3	21	48	F	Breast	Adjacent normal breast tissue	-	-	-	NAT	
D4	22	48	F	Breast	Adjacent normal breast tissue	-	-	-	NAT	
D5	23	46	F	Breast	Adjacent normal breast tissue	-	-	-	NAT	
D6	24	46	F	Breast	Adjacent normal breast tissue	-	-	-	NAT	
-	0	42	M	Adrenal gland	Pheochromocytoma (tissue marker)		-		Malignant	
